# Métastases intramédulaires d'un adénocarcinome pulmonaire: à propos d'un cas

**DOI:** 10.11604/pamj.2015.20.37.5500

**Published:** 2015-01-14

**Authors:** Hicham Naji-Amrani, Hicham Janah, Fatimazahra Sqalli, Hicham Souhi, Adil Zegmout, Hanane Elouazzani, Ismail Abderrahmani Rhorfi, Ahmed Abid

**Affiliations:** 1 Service de Pneumo-Phtisiologie, Hôpital Militaire d'Instruction Mohammed V, CHU Rabat, Maroc

**Keywords:** Métastases intramédullaires, IRM, Adénocarcinome bronchique, intramedullary metastasis, MRI, lung adenocarcinoma

## Abstract

Les métastases de la moelle épinière sont extrêmement rares. Elles surviennent chez 0,1 à 0,4% des patients cancéreux et représentent 1% de toutes les tumeurs de la colonne vertébrale et 1-3% des tumeurs intramédullaires. Le cancer du poumon est le primitif le plus fréquent. Nous rapportons le cas d'un patient de 51 ans, suivi pour un adénocarcinome pulmonaire et qui après la 1ère cure de chimiothérapie a développé des métastases intramédullaires et cérébrales. Une radiothérapie sur la moelle et le cerveau associée à une corticothérapie par voie générale ont été débutées. Le patient est décédé 3 mois après la survenue des métastases intramédullaires. A travers ce nouveau cas de métastases intarmédullaires d'un adénocarcinome pulmonaire et revue de la littérature, les auteurs insistent sur leur rareté ainsi que sur ses difficultés diagnostiques et thérapeutiques.

## Introduction

Les métastases intramédullaires (MIM) sont extrêmement rares. Seuls 300 cas de ont été rapportés dans la littérature [1]. Le cancer du poumon est le primitif le plus fréquent [2]. Les auteurs rapportent un nouveau cas de métastases de la moelle épinière d'un adénocarcinome pulmonaire en insistant sur leur rareté et ses difficultés diagnostiques et thérapeutiques.

## Patient et observation

**Figure 1:**
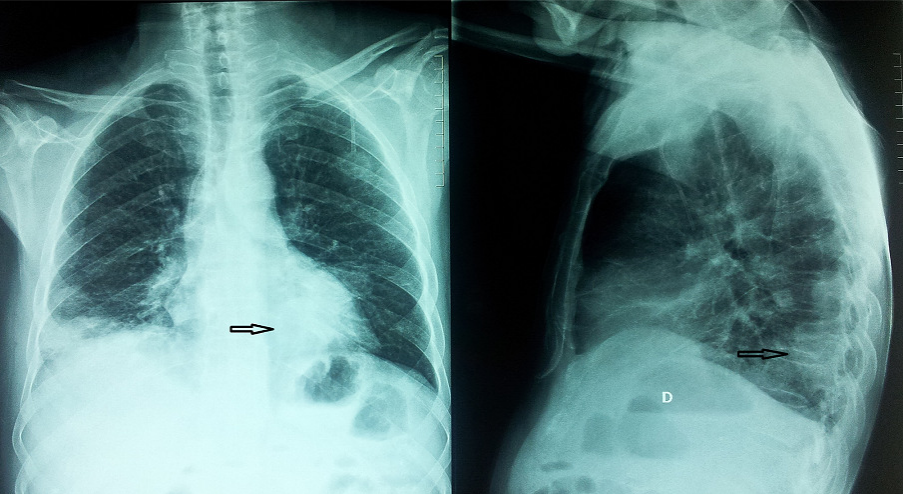
Radiographies du thorax vue de face et de profil montrant une opacité rétrocardiaque gauche

Il s'agit d'un Patient âgé de 51 ans, tabagique chronique de 30 paquets/année, accusant depuis un mois un syndrome bronchique trainant, une dyspnée d'effort et une altération de l’état général. L'examen clinique a noté la présence d'une adénopathie cervicale gauche. Les radiographies thoraciques de face et de profil ont montré une opacité ronde rétro-cardiaque gauche (Figure 1).

**Figure 2:**
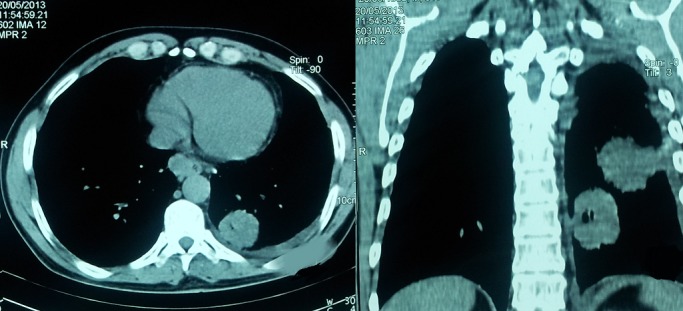
Scanner thoracique, deux masses tissulaires du poumon gauche

Le scanner thoraco-abdominal a objectivé deux masses tissulaires pulmonaires gauches, des adénopathies médiastinales, une pleurésie gauche et une masse surrénalienne gauche (Figure 2). La bronchoscopie a montré une muqueuse bronchique gauche d'aspect inflammatoire avec des éperons épaissis. L’étude anatomopathologique de la biopsie pulmonaire scannoguidée et de la biopsie exérèse de l'adénopathie cervicale a conclu à un adénocarcinome d'origine broncho-pulmonaire. Le scanner cérébral et la scintigraphie osseuse ont été sans anomalies. Le patient est mis sous chimiothérapie antimitotique à base de carboplatine-paclitaxel en attendant les résultats de la recherche de la mutation EGFR.

**Figure 3:**
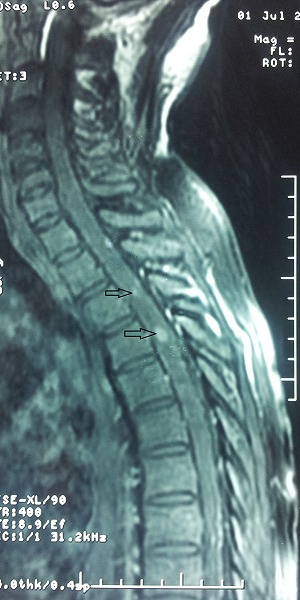
1^ère^ IRM médullaire, multiples anomalies du signal intramédullaire

**Figure 4:**
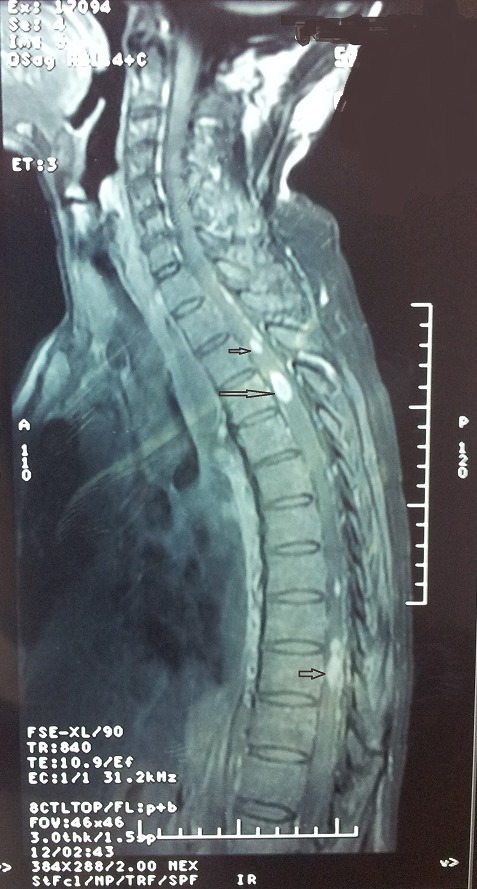
2^ème^ IRM médullaire, multiples métastases dorsales et lombaires

**Figure 5:**
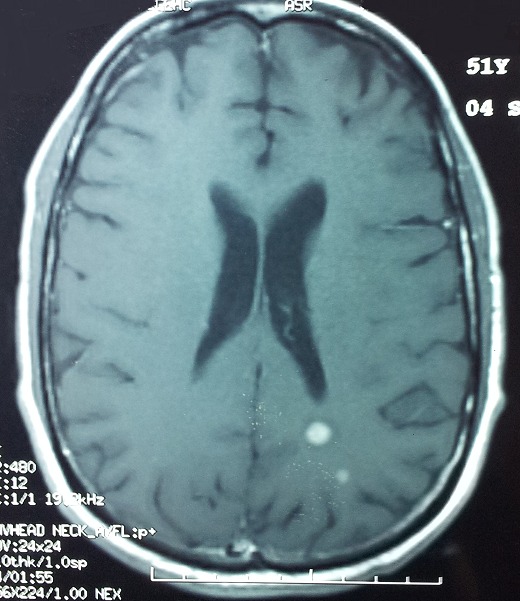
Métastases cérébrales

Lors de son hospitalisation pour la 2ème cure, le patient a développé des paresthésies du membre inferieur droit avec trouble de la marche et une paraparésie plus marquée à droite, l'examen neurologique a trouvé un syndrome pyramidal avec diminution de la sensibilité vibratoire. L'IRM médullaire a montré de multiples anomalies du signal intramédullaire en rapport avec une myélite (Figure 3). Le patient est mis sous traitement médical. Un mois après, la symptomatologie neurologique s'est aggravée par l'apparition des troubles sphinctériens, une deuxième IRM médullaire et cérébrale réalisée permettant de retenir le diagnostic de multiples métastases médullaires dorsales et lombaires associées à deux localisations cérébrales (Figure 4 et Figure 5)

Une radiothérapie sur la moelle et le cerveau associée à une corticothérapie par voie générale ont été débutées. Le patient est décédé 5 mois après le diagnostic du cancer.

## Discussion

Les métastases de la moelle épinière sont extrêmement rares. Elles surviennent chez 0,1 à 0,4% des patients cancéreux [3] et représentent 1% de toutes les tumeurs de la colonne vertébrale et 1-3% des tumeurs intramédullaires [1, 4]. Les MIM sont rarement isolées [5], souvent associées à d'autres métastases surtout cérébrales [6]. Le cancer du poumon est le primitif le plus fréquent (48% des cas) suivi du cancer du sein (16% des cas) [7].

Ces deux tumeurs primaires sont également des causes les plus fréquentes des métastases cérébrales, avec poumon (40%) et du sein (19%) [2]. La voie se dissémination mode des MIM est artérielle, ce qui pourrait expliquer la situation profonde des métastases, dans la moelle aux limites du territoire des artérioles médullaires. La voie périnerveuse est un mécanisme évoqué, mais discuté [8, 9].

La répartition des métastases intramédullaires dans la moelle tient essentiellement compte de la longueur du segment incriminé. Dans la plupart des séries, les MIM dorsales sont plus fréquentes, suivies des MIM cervicales et les lombaires, les localisations multiples n′étant pas exceptionnelles [10, 11]. Chez notre patient les métastases étaient dorsales et lombaires. La symptomatologie des MIM peut être inaugurale de la maladie cancéreuse, mais dans la plupart des cas, elle apparaît après la découverte du cancer primitif.

Généralement, Les MIM provoquent un œdème, une distorsion et compression du parenchyme de la moelle épinière, entraînant des douleurs et troubles sensitifs, suivis par une faiblesse, puis un dysfonctionnement sphinctérien [12]. Cependant, elles sont rarement asymptomatiques [13]. La détérioration rapide des symptômes neurologiques dans un délai moins d'un mois distingue les MIM des tumeurs intramédullaires primitives dont l’évolution des symptômes est généralement plus lente [4]. L'IRM est le seul examen à prescrire pour rechercher les MIM. Le bilan doit être le plus précis possible, avec de tout l'examen l'axe rachidien. Sur la région tumorale, il faut réaliser au moins 2 plans orthogonaux, le plus souvent avec des coupes sagittales et axiales pondérées T2 et T1 avant et après injection et une imagerie de tenseur de diffusion. Un examen cérébral complémentaire est recommandé [14, 15].

Les lésions prennent le contraste dans 98% des cas, sont multiples dans 20% des cas et sont associées à un œdème extensif s’étendant sur plus de 3 segments vertébraux. Des métastases osseuses associées sont à rechercher impérativement et sont fréquentes. Le diagnostic différentiel est essentiellement celui d'une tumeur primitive du SNC. Rykken [16] décrit deux signes séméiologiques orientant vers l'origine métastatique (hors néoplasie primitive du SNC): le « signe de l'anneau » (présence d'un rehaussement annulaire complet ou incomplet en dehors de la prise de contraste centrale) et le « signe de la flamme » (présence d'un rehaussement mal défini au pôle supérieur ou au pôle inférieur de la lésion, avec l'aspect d'une flammèche). Le signe de la flamme et le signe de l'anneau aident à différencier métastase intra-médullaire et néoplasie primitive.

D'autres diagnostics différentiels sont rapportés dans la littérature en cas d'images de MIM en plus des néoplasies primitives tels que l'hémangioblastome; les pathologies démyélinisantes du système nerveux central, une malformation artério-veineuse, la sarcoïdose et la myélite transverse [17]. Chez notre patient une myélite a été évoquée initialement et le diagnostic des MIM n'a été retenu qu'après aggravation clinique et apparition d'autres localisations métastatiques très évocatrices à la deuxième IRM.

La résection chirurgicale est indiquée quand le primitif est contrôlé avec une MIM localisée. Les objectifs de la chirurgie sont décompression de la moelle épinière, préservation de la fonction neurologique et de diagnostic histologique. La radiothérapie peut être utilisée comme un traitement curatif pour les patients en mauvais état général ou en tant que traitement adjuvant après chirurgie de réduction tumorale. Résection chirurgicale agressive n'entraîne pas de bons résultats fonctionnels, en raison de la nature infiltrative des MIM et sans un avantage de survie défini [18]. La radiothérapie fractionnée stéréotaxique ou radio-chirurgie est une option thérapeutique prometteuse [8, 19] mais encore peu répandue. Le bénéfice de la chimiothérapie est inconnu mais probablement dépend de la tumeur primaire [7]. Le pronostic des MIM d'origine pulmonaire reste péjoratif avec une médiane de survie de un mois [7, 19, 20].

Notre patient est décédé 3 mois après la survenue des MIM.

## Conclusion

Malgré leur rareté, les MIM doivent être toujours recherchées chez les patients atteints de cancer avec des troubles neurologiques d'installation rapide. L'IRM est l'examen clé pour le diagnostic. Le traitement n'est pas encore codifié. Le pronostic est sombre.
